# Age- and Gender-Specific Prevalence of Frailty and Its Outcomes in the Longevous Population: The Chinese Longitudinal Healthy Longevity Study

**DOI:** 10.3389/fmed.2021.719806

**Published:** 2021-08-02

**Authors:** Huai-yu Wang, Xiaozhen Lv, Jian Du, Guilan Kong, Luxia Zhang

**Affiliations:** ^1^National Institute of Health Data Science, Peking University, Beijing, China; ^2^School of Public Health, Peking University, Beijing, China; ^3^Clinical Research Division, Dementia Care and Research Center, Peking University Institute of Mental Health (Sixth Hospital), Beijing, China; ^4^Beijing Dementia Key Lab, National Clinical Research Center for Mental Disorders, Key Laboratory of Mental Health, Ministry of Health (Peking University), Beijing, China; ^5^Renal Division, Department of Medicine, Peking University First Hospital, Peking University Institute of Nephrology, Beijing, China; ^6^Advanced Institute of Information Technology, Peking University, Hangzhou, China

**Keywords:** frailty, prognosis, longevous population, age- and gender-disparity, all-cause mortality

## Abstract

**Background:** Frailty is an epidemic age-related syndrome addressing heavy burden to the healthcare system. Subject to the rarity, age-, and gender-specific prevalence of frailty and its prognosis among the longevous population remains under-investigated.

**Methods:** Based on the Chinese Longitudinal Healthy Longevity Study (CLHLS, 2008–2018), individuals aged ≥ 65 years having complete data of frailty were recruited. Modified Fried criteria (exhaustion, shrink, weakness, low mobility, and inactivity) were adopted to define pre-frailty (1–2 domains) and frailty (≥3 domains), respectively. The association between pre-frailty/frailty and adverse outcomes (frequent hospitalization, limited physical performance, cognitive decline, multimorbidity, and dependence) was analyzed using logistic regression models. The association between pre-frailty/frailty and mortality was analyzed using Cox proportional hazards models. Age- and gender-stratified analyses were performed.

**Results:** Totally, 13,859 participants aged 85.8 ± 11.1 years, including 2,056 centenarians, were recruited. The overall prevalence of pre-frailty and frailty were 54.1 and 26.3%, respectively. Only 5.0% of centenarians were non-frailty whereas 59.9% of the young-old (65–79 years) showed pre-frailty. Both pre-frailty and frailty were associated with the increased risk of multiple adverse outcomes, such as incident limited physical performance, cognitive decline and dependence, respectively (*P* < 0.05). Frail males were more vulnerable to the risk of mortality (hazard ratio [HR] = 2.3, 95% confidence interval [CI], 2.1–2.6) compared with frail females (HR = 1.9, 95%CI, 1.7–2.1). The strongest association between frailty and mortality was observed among the young-old (HR = 3.6, 95%CI, 2.8–4.5). Exhaustion was the most common domain among patients with pre-frailty (74.8%) or frailty (83.2%), followed by shrink (32.3%) in pre-frailty and low mobility (83.0%) in frailty. Inactivity among females aged 65–79 years showed the strongest association with the risk of mortality (HR = 3.50, 95%CI, 2.52–4.87).

**Conclusion:** A huge gap exists between longer life and healthy aging in China. According to the age- and gender-specific prevalence and prognosis of frailty, the strategy of frailty prevention and intervention should be further individualized.

## Introduction

Frailty is a series of age-related clinical conditions showing the deterioration of strength and physiologic malfunction (1–5). It is strongly associated with the susceptibility of stressors, manifested as the vulnerability to multiple diseases and the delayed recovery (1, 3, 4). Studies in Europe indicated that frail individuals cost three to five times of healthcare services compared with non-frail ones (6, 7). Thus, frailty significantly increases the pressure to the healthcare system to cope with the challenge of aging. Since frailty is a biological syndrome of aging, the prevalence of frailty grows in keeping with the rapid expansion of the aging population (4–6). Previous studies reported the prevalence of frailty among the population aged 60 years or older as 4.0–59.1% in high-income countries (8) and 3.9–65.3% in low- and middle-income countries (9–11). However, subject to the rarity of longevous population and the difficulty of their long-term response to the follow-up, the burden of frailty among the longevous population remains under-investigated. Recently, Herr et al. (12) explored the prevalence of frailty among 1,253 centenarians in five high-income countries and reported the prevalence of frailty as 64.7% (12). Although all participants included in this study were from developed countries, Herr et al. (12) reported the association between residence of country and the risk of frailty among centenarians. It further stressed the necessity to explore the burden of frailty in different regions.

As the largest developing country, China shows unique process of aging due to the one-child policy and rapid development of social economy (13, 14). Owing to the size and globalization, the evaluation of the burden of frailty in China has potentially scientific implications to the global strategy to promote healthy aging (15). According to the data from the National Bureau of Statistics, there were 176 million people aged≥65 years in China in 2019 (1), while this number was predicted as 400 million (26.9% of the total population) in 2050 and 150 million of them were aged 80 years or older (13). The number of the oldest population (≥80 years) increases roughly 10% annually in China and around one quarter of the global oldest population will live in China by 2050 (16). However, a huge gap exists between long life expectancy and healthy aging. Previous studies reported the prevalence of frailty as 3.1–25.0% in China, while few of them investigated the prevalence and the outcomes among the large sample size of the oldest people with a long period of follow-up (9–11, 17–21). As an essential clinical manifestation among the aging people, evaluating the burden of frailty among the longevous population in China, which should contain the insight of both the prevalence and the outcomes, would supplement the insight of global frailty and evidence the modification of the frailty management among the oldest population.

The Chinese Longitudinal Healthy Longevity Surveys (CLHLS) is an ongoing nationally representative cohort drawing data from the longevous population (22–25). Till 2018, 67.4% of participants in CLHLS were people aged 80 years or older, and the CLHLS had interviewed over 20 thousand person-times of centenarian, nonagenarian and octogenarian, respectively (23). Based on this precious cohort, the present study investigated the prevalence of pre-frailty and frailty among the community-dwelling population with advanced age. The association between pre-frailty/frailty and multiple adverse outcomes was also investigated. Given the disparity of frailty between genders and age groups (1, 4), gender- and age-stratified analyses were performed.

## Methods

### Population

The CLHLS was conducted in a randomly-selected half of the counties and cities in 22 of the 31 provinces, which covering 85% of population of China (22–26). The CLHLS recruited a large sample size of centenarians and the approximately equal numbers of nonagenarians, octogenarians, and young-old (aged 65–79 years) in both genders living in the same area with the centenarians so as to ensure the representativeness (23). From 1998 on, the interview was conducted every 3–4 years using the structured questionnaires. Detailed description of CLHLS could be found elsewhere (22–26).

The present study was conducted based on the 2008 cohort of CLHLS including interviews of 2008, 2011, 2014, and 2018 (23, 26). A total of 16,954 participants were included in the cohort, 2,710 of them were excluded because of the absence of frailty-related data. Another 385 individuals aged <65 years were also excluded. Ultimately, 13,859 participants aged ≥ 65 years and having complete data on frailty were included in the current analyses.

The CLHLS was approved by the Research Ethics Committee of Peking University (IRB00001052-13074). All participants provided written informed consent.

### Covariates

Age groups was defined as 65–79, 80–89, 90–99, and ≥100 years. The level of education was categorized as illiteracy, primary school, and middle school or above in accordance with the years being educated. The levels of household income were recorded as quartiles. Status of smoke and drink were recorded as never, past, and current. Body mass index (BMI, kg/m^2^) was calculated as weight divided by height square and categorized into normal (18.5–23.9 kg/m^2^), underweight (<18.5 kg/m^2^), overweight (24.0–27.9 kg/m^2^), and obesity (≥28.0 kg/m^2^). Activities of daily living (ADL) was evaluated through six daily activities (eating, dressing, transferring, using the toilet, bathing, and continence). Impaired ADL was defined if the participant need help for one or more activities; dependency was defined if the participant could not complete one or more activities with or without help. Self-reported comorbidity was recorded including hypertension, diabetes, heart disease, cerebrovascular disease, chronic pulmonary disease (chronic bronchitis, emphysema, or asthma), eye disease (cataract or glaucoma), cancer, Parkinson's disease, dementia, mental disease, arthritis, gastrointestinal ulcer, hepatitis, and others.

### Criteria for Frailty

The modified Fried criteria was adopted to define the frailty status (2, 12, 17). Five domains including exhaustion, shrink, weakness, low mobility, and inactivity were evaluated using self-report data.

Exhaustion was defined if the participant answered “always,” “often,” or “sometimes” to either of the questions “I felt old and useless” or “I felt everything I did was an effort” (17, 27). Shrink was defined as BMI < 18.5 kg/m^2^ (12, 17, 28). Weakness was defined if the participant failed to lift a bag weighting 5 kg (12, 28). Low mobility was defined if the participant failed to walk for 1 km (29). Inactivity was defined if the participant did the following activities 1 time per week or less: housework, outside activity, gardening, keeping a pet, livestock breeding, playing cards or moh-jong, and social activity (27).

Participant meeting 1–2 domains was defined as pre-frailty. Participant meeting ≥3 domains was defined as frailty. The prevalence of pre-frailty and frailty was defined according to the 2008 interview.

### Outcomes

Outcomes were defined using the data of the 2011, 2014, and 2018 waves. Frequent hospitalization was defined if the participant been in hospital ≥3 times due to severe illness during the past 2 years before the interview. Incident limited physical performance was defined if the participant completed the objective performance-based tests at baseline but failed during the follow-up (22). Incident cognitive decline was defined if the participant had the MMSE score ≥ 23 at baseline but <23 during the follow-up (22). Incident multimorbidity was defined if the participant reported 0–2 comorbidities at baseline while ≥3 comorbidities during the follow-up (30). Incident dependency was defined if the participant showed normal or impaired ADL at baseline but being dependency during the follow-up. All-cause mortality was recorded. The median duration of follow-up was 55 (IQR 25–95) months.

### Statistics

Demographic characteristics (age, gender), socioeconomic characteristics (education, household income), lifestyles (smoke, drink), physical health status (BMI, comorbidity count) and follow-up duration were presented by the status of frailty at baseline (non-frailty, pre-frailty, and frailty). Chi-square tests, oneway ANOVA, and Kruskal–Wallis tests were applied for the comparison of categorical, normal distributed continuous, and skewed distributed continuous variables, respectively. The prevalence of frailty domains (exhaustion, shrink, weakness, low mobility, and inactivity) was analyzed. Age- and gender-stratified analyses of frailty status and frailty domains were calculated, respectively.

Multivariate logistic regression models were adopted to separately analyze the association between the frailty status (non-frailty, pre-frailty, and frailty) and the risk of frequent hospitalization, incident limited physical performance, incident cognitive decline, incident multimorbidity, and incident dependency. Covariates including age, gender, education, household income, smoke, and comorbidity count were adjusted. Results were presented as odds ratio (OR) with 95% confidence interval (CI). Gender-stratified analyses were performed.

Cox proportional hazards regression models were adopted to analyze the association between frailty status (non-frailty, pre-frailty, and frailty), domains of frailty (exhaustion, shrink, weakness, low mobility, and inactivity) and the risk of all-cause mortality, respectively. Covariates including education, household income, smoke, and comorbidity count were adjusted. Age- and gender-stratified analyses were performed. Hazard ratio (HR) and 95% CI was calculated.

All analyses were two tailed and *P* < 0.05 was considered to be statistical significance. Stata version 16.0 (Stata Corp LP, College Station, TX, USA) were used for all statistical analyses.

## Results

### Population Characteristics

A total of 13,859 participants aged 85.8 ± 11.1 (range 65–116) years were included. Among them, 2,056 (14.8%) were centenarians and 3,690 (26.6%) were nonagenarians. Totally, 59.2% of participants were in rural area and 59.7% of participants were illiteracy. Underweight (31.4%) was more common compared with obesity (2.8%) among the studied population. Altogether 27.4, 31.7, 9.8, and 8.0% of participants showed limited physical performance, cognitive decline, dependency and multimorbidity at baseline, respectively (Table 1).

**Table 1:** Comparison of characteristics among participants in different status of frailty at baseline.

**Characteristics**	**Overall**	**Non-frailty**	**Pre-frailty**	**Frailty**	***P-*value**
In total, No. (%)	13,859 (100.0)	2,715 (19.6)	7,497 (54.1)	3,647 (26.3)	
Follow-up duration, IQR, month	55 (25,95)	71 (42,118)	65 (30,106)	28 (14,54)	<0.001
Mean age, years, mean (SD)	85.8 (11.1)	79.2 (10.0)	84.2 (10.5)	93.8 (8.2)	<0.001
**Age group, No. (%)**					<0.001
65–79 years	4,180 (30.2)	1,475 (54.3)	2,505 (33.4)	200 (5.5)	
80–89 years	3,933 (28.4)	729 (26.9)	2,440 (32.6)	764 (21.0)	
90–99 years	3,690 (26.6)	408 (15.0)	1,811 (24.2)	1,471 (40.3)	
≥100 years	2,056 (14.8)	103 (3.8)	741 (9.9)	1,212 (33.2)	
**Gender, No. (%)**					<0.001
Male	6,252 (45.1)	1,538 (56.7)	3,593 (47.9)	1,121 (30.7)	
Female	7,607 (54.9)	1,177 (43.4)	3,904 (52.1)	2,526 (69.3)	
**Regions, No. (%)**					<0.001
Urban	5,648 (40.8)	1,295 (47.7)	2,814 (37.5)	1,539 (42.2)	
Rural	8,211 (59.2)	1,420 (52.3)	4,683 (62.5)	2,108 (57.8)	
**Education, No. (%)**					<0.001
Illiteracy	8,279 (59.7)	1,110 (40.9)	4,421 (59.0)	2,748 (75.4)	
Primary school	4,082 (29.5)	1,081 (39.8)	2,311 (30.8)	690 (18.9)	
Middle school or above	1,498 (10.8)	524 (19.3)	765 (10.2)	209 (5.7)	
**Household income, No. (%)**					<0.001
Quartile 1	4,152 (30.0)	632 (23.3)	2,492 (33.2)	1,028 (28.2)	
Quartile 2	2,980 (21.5)	582 (21.4)	1,576 (21.0)	822 (22.5)	
Quartile 3	3,859 (27.8)	834 (30.7)	2,042 (27.2)	983 (27.0)	
Quartile 4	2,868 (20.7)	667 (24.6)	1,387 (18.5)	814 (22.3)	
**Smoke, No. (%)**					<0.001
Never	9,095 (65.7)	1,574 (58.0)	4,747 (63.3)	2,774 (76.2)	
Past	2,233 (16.1)	492 (18.1)	1,224 (16.3)	517 (14.2)	
Current	2,524 (18.2)	649 (23.9)	1,524 (20.3)	351 (9.6)	
**Drink, No. (%)**					<0.001
Never	9,484 (68.5)	1,681 (61.9)	5,019 (67.0)	2,784 (76.4)	
Past	1,912 (13.8)	371 (13.7)	1,055 (14.1)	486 (13.3)	
Current	2,454 (17.7)	662 (24.4)	1,420 (19.0)	372 (10.2)	
**BMI, No. (%)**					<0.001
Normal	7,557 (54.5)	2,086 (76.8)	4,044 (54.0)	1,427 (39.1)	
Underweight	4,352 (31.4)	0 (0.0)	2,418 (32.3)	1,934 (53.0)	
Overweight	1,563 (11.3)	516 (19.0)	834 (11.1)	213 (5.8)	
Obesity	387 (2.8)	113 (4.2)	201 (2.7)	73 (2.0)	
**Limited physical performance, No. (%)**					<0.001
No	10,057 (72.6)	2,506 (92.4)	6,189 (82.6)	1,362 (37.4)	
Yes	3,798 (27.4)	207 (7.6)	1,308 (17.5)	2,283 (62.6)	
**Cognitive decline, No. (%)**					<0.001
No	9,464 (68.4)	2,430 (89.5)	5,723 (76.4)	1,311 (36.0)	
Yes	4,383 (31.7)	284 (10.5)	1,773 (23.7)	2,326 (64.0)	
**ADL, No. (%)**					<0.001
Normal	11,709 (84.5)	2,653 (97.7)	7,020 (93.6)	2,036 (55.8)	
Impaired	791 (5.7)	42 (5.3)	261 (3.5)	488 (13.4)	
Dependency	1,359 (9.8)	20 (0.7)	216 (2.9)	1,123 (30.8)	
**Count of comorbidity, No**. (**%)**					<0.001
None	5,888 (45.6)	1,241 (48.0)	3,274 (46.9)	1,373 (41.0)	
1–2 comorbidities	5,984 (46.4)	1,165 (45.1)	3,181 (45.6)	1,638 (48.9)	
≥3 comorbidities	1,036 (8.0)	178 (6.9)	522 (7.5)	336 (10.0)	

*SD, standard deviation; BMI, body mass index; ADL, activity of daily live*.

As to participants with pre-frailty, although they were at high-risk of frailty, 1,524 (20.3%) and 1,420 (19.0%) pre-frail individuals were current smokers and drinkers, respectively. The highest proportion of illiteracy (75.4%) and the lowest proportion of obesity (2.0%) were observed among the frail population. Limited physical performance (62.6%), impaired ADL (13.4%), dependency (30.8%) cognitive decline (64.0%), and having comorbidities (58.9%) were more common among frail population compared with non-frail ones (Table 1).

### Prevalence of Pre-frailty and Frailty

**Figure 1:**
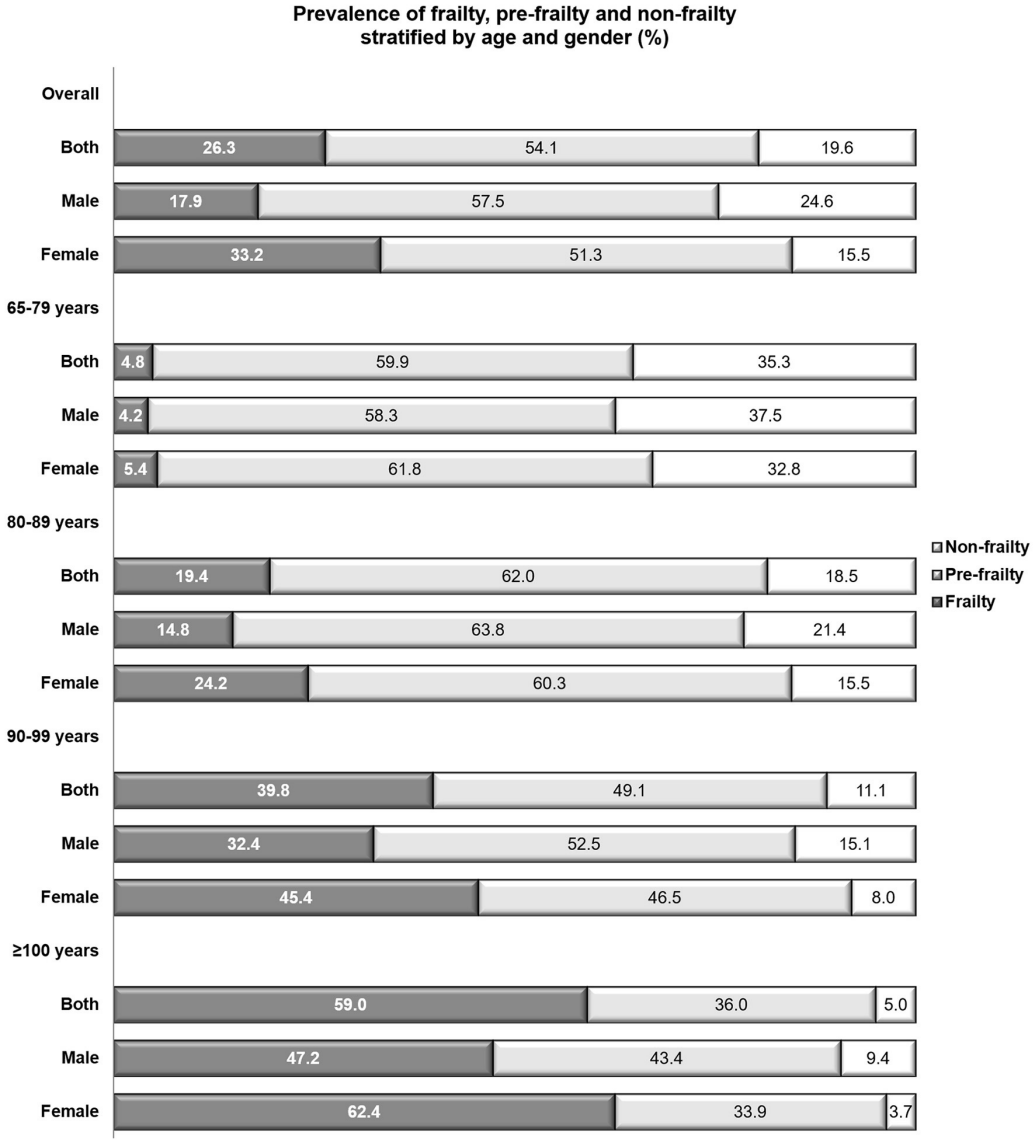
Prevalence of pre-frailty, frailty and non-frailty, age-, and gender-stratified.

In accordance with the modified Fried criteria, 7,497 participants with pre-frailty and 3,647 participants with frailty were identified, respectively. The overall prevalence of pre-frailty and frailty was 54.1 and 26.3%, respectively (Table 1). Nearly doubled prevalence was observed among females compared with males (females vs. males: 33.2 vs. 17.9%) while slightly higher prevalence of pre-frailty was observed among males in contrast to females (males vs. females: 57.5 vs. 51.3%; Figure 1).

According to the age-stratified analyses, the prevalence of frailty among the young-old (<80 years), octogenarians, nonagenarians and centenarians was 4.8, 19.4, 39.9, and 59.0%, respectively. The prevalence of pre-frailty peaked among the octogenarians (62.0%) and decreased with aging (centenarians: 36.0%) (Figure 1). The gender-stratified analyses showed that females were with higher prevalence of frailty compared with males in all age groups and males showed higher prevalence of pre-frailty compared with females in groups aged ≥80 years. The highest prevalence of pre-frailty was observed among males aged 80–89 years (63.8%) and the highest prevalence of frailty was observed among females aged ≥ 100 years (62.4%) (Figure 1).

### Domains of Frailty

Among pre-frail population, exhaustion (74.8%), shrink (32.3%), and inactivity (14.6%) were the most frequent domains. The prevalence of exhaustion decreased with age while that of shrink, weakness, low mobility, and inactivity increased with age. Females were more likely to be shrink in all age groups and males were obviously inactive except for centenarians (Supplementary Table 1).

Among frail population, exhaustion (83.2%), low mobility (83.0%), and weakness (82.5%) were the most frequent domains. The prevalence of exhaustion peaked among the young-old (<80 years, 91.0%). The prevalence of low mobility (88.8%) exceeded that of exhaustion (78.7%) and became the most common domains among the centenarians. Gender-specific analyses showed that shrink, weakness, and low mobility were more common among females in all age groups whereas inactivity was more common among males (Supplementary Table 1).

### Pre-frailty, Frailty, and Adverse Outcomes

After adjusting for confounders, pre-frailty was significantly associated with the risk of limited physical performance (OR = 1.2, 95%CI, 1.1–1.4), incident cognitive decline (OR = 1.4, 95%CI, 1.1–1.7) and dependency (OR = 1.4, 95%CI, 1.2–1.7), respectively. Frailty was strongly associated with nearly doubled risk of frequent hospitalization and more than five times increased risk of incident limited physical performance, incident cognitive decline, and incident dependency, respectively (Table 2).

**Table 2:** Association between pre-frailty/frailty and the risk of multiple adverse outcomes.

**Outcomes**	**Case no**.	**Adjusted OR^a^ (95% CI)**	***P*-value**
**Frequent hospitalization**			
Non-frailty	138	Ref.	Ref.
Pre-frailty	318	1.2 (1.0–1.5)	0.090
Frailty	68	1.9 (1.3–2.7)	0.001
**Incident limited physical performance**			
Non-frailty	2,025	Ref.	Ref.
Pre-frailty	5,422	1.2 (1.1–1.4)	0.004
Frailty	1,344	5.2 (2.9–9.2)	<0.001
**Incident cognitive decline**			
Non-frailty	402	Ref.	Ref.
Pre-frailty	1,277	1.4 (1.1–1.7)	0.003
Frailty	307	5.2 (3.0–9.0)	<0.001
**Incident multimorbidity^b^**			
Non-frailty	447	Ref.	Ref.
Pre-frailty	990	1.3 (1.0–1.7)	0.050
Frailty	401	17.3 (5.3–56.1)	<0.001
**Incident dependence**			
Non-frailty	327	Ref.	Ref.
Pre-frailty	1,142	1.4 (1.2–1.7)	0.001
Frailty	448	5.7 (3.8–8.7)	<0.001

a *Adjusted for: age, gender, education, household income, smoke status, and comorbidity count at baseline*.

b *Adjusted for: age, gender, education, household income, and smoke status*.

*OR, odds ratio; CI, confidence interval*.

Subject to the number of incident cases, gender-stratified analyses were only performed regarding outcomes of incident limited physical performance, incident cognitive decline, and incident dependency. Females with pre-frailty, instead of males, were with the increased risk of incident limited physical performance (OR = 1.3, 95%CI, 1.1–1.6), cognitive decline (OR = 1.4, 95%CI, 1.1–1.9), and dependency (OR = 1.7, 95%CI, 1.3–2.2). Frailty was significantly associated with the risk of these adverse outcomes in both genders and showed more intensive influence to males compared with females (*P* < 0.001; Supplementary Table 2).

### Frailty Status and All-Cause Mortality

**Figure 2:**
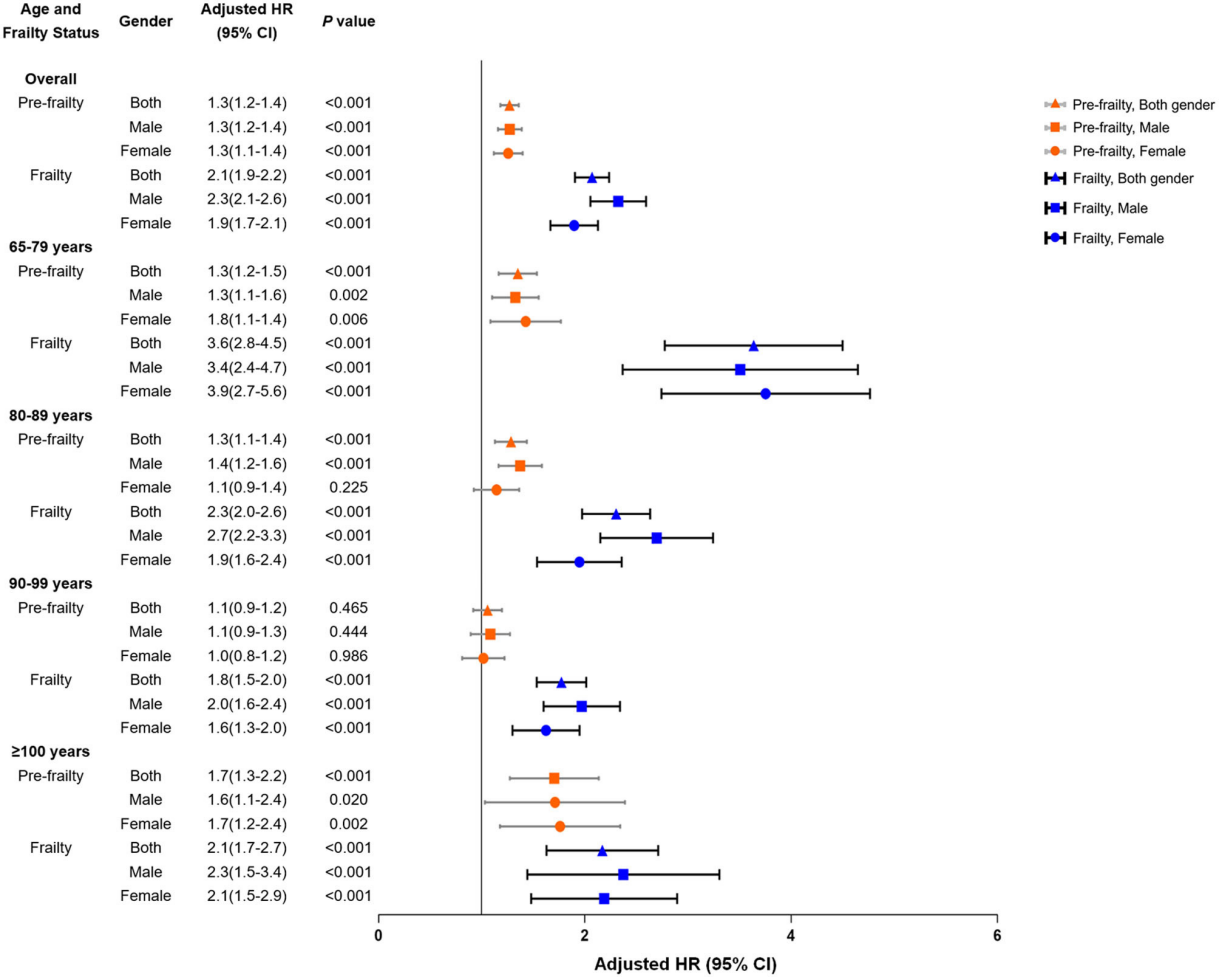
All-cause mortality for pre-frailty and frailty at baseline, stratified by age and gender. Adjusted for education, household income, smoke status, and comorbidity count at baseline.

After adjusting for confounders, both pre-frailty and frailty were strongly correlated to the increased risk of mortality (*P* < 0.001). The association between pre-frailty and the risk of mortality peaked among centenarians (HR = 1.7, 95%CI, 1.3–2.2). The strongest association between frailty and mortality was observed among the group aged 65–79 years (HR = 3.6; 95% CI, 2.8–4.5). Among the age groups ≥ 80 years, frail males were much vulnerable to the risk of mortality in contrast to frail females (Figure 2).

### Domains of Frailty and All-Cause Mortality

**Figure 3:**
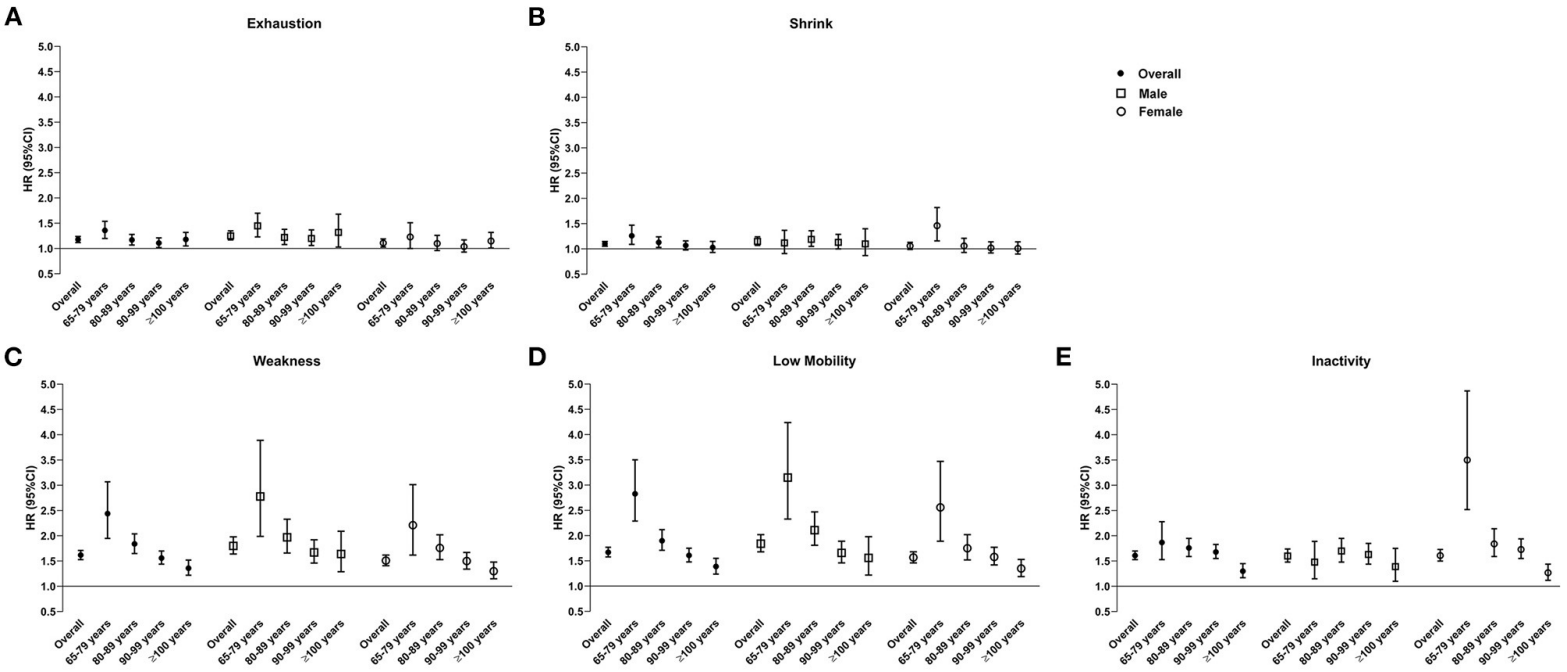
Association between domains of frailty [**(A)** exhaustion, **(B)** shrink, **(C)** weakness, **(D)** low mobility, and **(E)** inactivity] and the risk of all-cause mortality, age-, and gender-stratified. Adjusted for: education, household income, smoke status, and comorbidity count at baseline.

All of the domains of frailty were significantly associated with the increased risk of all-cause mortality (Figure 3; Supplementary Table 3) According to the age- and gender-stratified analyses, the association between exhaustion and the risk of mortality showed U-shaped trend with aging in both genders and was more influential to males in contrast to females (Figure 3A). Shrink was significantly associated with the increased risk of mortality among females aged 65–79 years (HR = 1.46, 95%CI, 1.16–1.82) and males aged 80–89 years (HR = 1.19, 95%CI, 1.05–1.36) (Figure 3B; Supplementary Table 3). Males aged 65–79 years with weakness (HR = 2.78, 95%CI, 1.99–3.89) and with low mobility (HR = 3.15, 95%CI, 2.33–4.24) were with the highest risk of mortality in contrast to females and males in other age groups (Figures 3C,D; Supplementary Table 3). The strongest association between inactivity and the risk of mortality was observed among females aged 65–79 years (HR = 3.50, 95%CI, 2.52–4.87) (Figure 3E; Supplementary Table 3).

## Discussion

Based on the nationwide cohort study of the longevous population, the present study reported the prevalence of pre-frailty and frailty among the population with a mean age of 85 years, which were 54.1 and 26.3%, respectively. Females were predominant among frail population in all age groups whereas males were dominant among pre-frail individuals aged ≥ 80 years. Both pre-frailty and frailty were strongly associated with multiple adverse outcomes. Males and the young-old (<80 years) were the most susceptible to the risk of mortality. Although all of the domains were significantly associated with adverse outcomes, physical deficits including weakness, low mobility and inactivity showed stronger association with the risk of mortality compared with exhaustion and shrink. The current results supplemented previous data on the prevalence of frailty among the longevous population and provided clues to develop the strategy of frailty treatment. Intensified prevention and treatment of frailty should be applied in China. The gender-specific strategy should be developed.

Healthy aging is an important goal of the public health in the 21st century. However, a huge gap exists between longer life and healthy aging. The present study indicated the high prevalence of pre-frailty and frailty among the Chinese longevous population, which is consistent with the results from 1,253 centenarians in the 5-COOP countries (Japan, France, Switzerland, Sweden, and Denmark) (12). It demonstrated that the heavy burden of frailty among the longevous population was globally substantial. Notably, only 5% of centenarians and 11.1% of nonagenarians were non-frailty in the present study. Similar results were observed among centenarians in the 5-COOP countries (12). Additionally, the present results comprehensively demonstrated the association between pre-frailty, frailty, and the risk of multiple adverse outcomes. It implies not only the high consumption of healthcare resources of the frail elderly, but also the suffering of patients themselves. Hence, the epidemic of frailty could be considered as one of the great barriers of healthy aging. The healthcare system in the developing countries, such as China, should be changed as soon as possible so as to better prevent and treat frailty and cope with the challenge of aging.

The current results found the strongest association between frailty and the risk of mortality among the young-old although the prevalence of frailty was the lowest in this group. Similar results were reported in previous studies (31–34). Dupre et al. (35) investigated frailty and the type of death among aged people in China, which was categorized according to the bedridden and suffering before death. They found that the young-old (65–79 years) with any levels of frailty showed the longest period of bedridden with suffering before death in contrast to other older groups. Abraham et al. (36) analyzed suffering at the end of life among individuals without acknowledged physical distress and found that the mean age of moderate-to-severe suffering group was significantly younger than no-to-mild suffering group (65 vs. 75 years, *P* < 0.05). It indicated that a distinguishing mechanism of deathbed or mortality might exist among the young-old population compared with those with advanced age. However, to our knowledge, rare studies explored the underlying mechanisms. In contrast to the chronological age, frailty is a much specific indicator of physical and biological senescence (27) and shows significantly gender-specific association with multiple adverse outcomes. Although the underlying mechanism of frailty and premature death remains to be explored, the present results provide validate results to strongly stress the importance to prevent and treat frailty among the young-old population.

The gender-specific prevalence of frailty and its association between mortality were found in the present study, which is consistent with previous studies (37, 38). Corbi et al. (37) investigated the inter-relationship between gender, frailty, and the 10-year survival among 1,284 adults with a mean age of 74.2 years. Although more females with frailty were found compared with males (50.3 vs. 29.5%), female gender was associated with the reduced risk of mortality (HR = 0.43, 95%CI: 0.299–0.561). Zhang et al. (38) reported the higher prevalence of frailty among females (8.8 vs. 5.4%) and the higher mortality among frail males (22.5 vs. 8.5%). In addition to the community-dwelling population, the gender difference of prevalence of frailty was also found among patients with HIV-infection (39). It strongly suggests the necessity to develop the gender-specific strategies for management and prevention of frailty. Previously, Serra-Prat et al. (40) conducted a randomized controlled trial and demonstrated the effectiveness of an intervention focused on physical activity and nutrition to prevent frailty in pre-frail population. However, according to the present results of gender-difference of frailty, the gender-specific effectiveness of intervention on activity and nutrition should be further investigated. According to the study of Zhang et al. (38), heart disease and nephritis were the leading causes of death among the frail males and females, respectively. Komici et al. (41) reviewed the cardioprotective effects of dietary phytochemicals and reported the gender-differences of the adsorption, distribution, metabolism, and elimination of dietary phytochemicals. For instance, a better effect of quercetin against atherosclerosis was found among females, which might be influenced by the better absorption among females, while a lower kidney elimination of the conjugated phenolic compounds was found among females. In sum, the impact of gender on the pathogenesis of frailty should be further explored and the development of the gender-specific strategies for prevention and management of frailty should fully consider the epidemiological factors and the underlying mechanisms of the gender-differences.

Heterogeneity is one of the major characteristics of the natural course of frailty, it increases the challenge of early management of frailty (1). Previous studies investigating the phenotype and progression of frailty were mainly from Caucasians in developed countries. Results from the Women's Health and Aging Study II (42) (included 420 females aged 70–79 years) indicated weakness as the initial manifestation whereas results from the Longitudinal Aging Study Amsterdam and the Netherlands (LASA, *n* = 1,440) and the Invecchiare in Chianti, aging in the Chianti area (InCHIANTI, *n* = 998) Study showed that exhaustion was the first manifestation of frailty (43). According to the present results, exhaustion was the predominant domain among pre-frail population while the prevalence of physical deficits, such as weakness and low mobility, obviously increased among the frail population. It showed that the progression of frailty among the Chinese longevous population was from exhaustion to physical deficits. It is consistent with results from LASA and InCHIANTI study. Additionally, the present study demonstrated the association between domains of frailty and the risk of all-cause mortality. Although weakness, low mobility and inactivity emerged later and dominated in frailty among the longevous population, these physical deficits showed significantly stronger association with the risk of mortality compared with exhaustion. Our results indicated the importance of prevention of exhaustion among the aging population and stressed the prevention and treatment of physical deficits among pre-frail and frail population. Family- and community-based system of multicomponent training, such as exercise and social activities, would be feasible and beneficial (44). Besides, high prevalence of shrink among females and inactivity among males indicated the necessity of gender-specific strategy of frailty management. Caregivers should enhance the nutrition supplement especially among females and improve the physical activity especially among males.

The present study has limitations. First, data of comorbidity was self-reported. Influenced by awareness, the status of multimorbidity might be under-estimated. Second, given the heterogeneity of existing tools for frailty screening (1), studies generating and using other tools to quantify frailty are still expected although the criteria adopted in the present study has been widely used. Third, the determinants of mortality in addition to the status of frailty were not investigated in the present study subject to the availability of data. Fourth, the possibility of residual confounding exists.

In conclusion, frailty is prevalent among the longevous population in China. The association between pre-frailty, frailty, and multiple adverse outcomes emphasized the importance to prevent and treat frailty in the elderly. Given the disparity of frailty between genders and age groups, gender-, and age-specific strategies should be developed to prevent the adverse outcomes.

## Data Availability Statement

Publicly available datasets were analyzed in this study. This data can be found here: the datasets analyzed during the current study are available in the Chinese Longitudinal Healthy Longevity Surveys repository, https://opendata.pku.edu.cn/dataverse/CHADS.

## Ethics Statement

The CLHLS was approved by the Research Ethics Committee of Peking University (IRB00001052-13074). All participants provided written informed consent. The patients/participants provided their written informed consent to participate in this study.

## Disclosure

All authors listed meet the criteria for authorship, have approved the submission and release the copyright for publication.

## Author Contributions

HYW designed the study, analyzed the data, interpreted the results, and wrote the manuscript. XL advised the methodology and interpreted the results. JD and GK revised the manuscript. LZ supervised the study, revised the manuscript, and is the study garantor. All authors contributed to the article and approved the submitted version.

## Conflict of Interest

The authors declare that the research was conducted in the absence of any commercial or financial relationships that could be construed as a potential conflict of interest.

## Publisher's Note

All claims expressed in this article are solely those of the authors and do not necessarily represent those of their affiliated organizations, or those of the publisher, the editors and the reviewers. Any product that may be evaluated in this article, or claim that may be made by its manufacturer, is not guaranteed or endorsed by the publisher.
