# Supplementary material for: Age- and Gender-Specific Prevalence of Frailty and Its Outcomes in the Longevous Population: The Chinese Longitudinal Healthy Longevity Study
Source: Front Med (Lausanne). 2021 Aug 2;8:719806. doi: 10.3389/fmed.2021.719806 (PMC8365226; doi:10.3389/fmed.2021.719806)
Supplement: Supplementary file 1 [file Table_1.DOCX]

**Supplementary Table 1. Prevalence of frailty domains, age- and gender-stratified.**

| Groups | Exhaustion | Shrink | Weakness | Low Mobility | Inactivity |
| --- | --- | --- | --- | --- | --- |
| *Pre-frailty* |  |  |  |  |  |
| Overall, No. (%) |  |  |  |  |  |
| Both | 5610(74.8) | 2418(32.3) | 497(6.6) | 500(6.7) | 1097(14.6) |
| Male | 2707(75.3) | 1036(28.8) | 162(4.5) | 218(6.1) | 591(16.5) |
| Female | 2903(74.4) | 1382(35.4) | 335(8.6) | 282(7.2) | 506(13.0) |
| 65-79 years, No. (%) |  |  |  |  |  |
| Both | 2084(83.2) | 654(26.1) | 59(2.4) | 71(2.8) | 168(6.7) |
| Male | 1048(81.5) | 323(25.1) | 15(1.2) | 29(2.3) | 132(10.3) |
| Female | 1036(85.0) | 331(27.2) | 44(3.6) | 42(3.5) | 36(3.0) |
| 80-89 years, No. (%) |  |  |  |  |  |
| Both | 1859(76.2) | 805(33.0) | 148(6.1) | 171(7.0) | 306(12.5) |
| Male | 963(75.5) | 383(30.0) | 47(3.7) | 78(6.1) | 204(16.0) |
| Female | 896(77.0) | 422(36.3) | 101(8.7) | 93(8.0) | 102(8.8) |
| 90-99 years, No. (%) |  |  |  |  |  |
| Both | 1220(67.4) | 676(37.3) | 188(10.4) | 147(8.1) | 417(23.0) |
| Male | 569(68.8) | 265(32.0) | 73(8.8) | 72(8.7) | 201(24.3) |
| Female | 800(83.3) | 411(41.8) | 115(11.7) | 75(7.6) | 216(22.0) |
| ≥100 years, No. (%) |  |  |  |  |  |
| Both | 447(60.3) | 283(38.2) | 102(13.8) | 111(15.0) | 206(27.8) |
| Male | 127(62.3) | 65(31.9) | 27(13.2) | 39(19.1) | 54(26.5) |
| Female | 320(59.6) | 218(40.6) | 75(14.0) | 72(13.4) | 152(28.3) |
| *Frailty* |  |  |  |  |  |
| Overall, No. (%) |  |  |  |  |  |
| Both | 3036(83.2) | 1934(53.0) | 3008(82.5) | 3026(83.0) | 2638(72.3) |
| Male | 941(83.9) | 545(48.6) | 848(75.7) | 900(80.3) | 844(75.3) |
| Female | 2095(82.9) | 1389(55.0) | 2160(85.5) | 2126(84.2) | 1794(71.0) |
| 65-79 years, No. (%) |  |  |  |  |  |
| Both | 182(91.0) | 93(46.5) | 160(80.0) | 163(81.5) | 113(56.5) |
| Male | 86(92.5) | 42(45.2) | 66(71.0) | 75(80.7) | 62(66.7) |
| Female | 96(89.7) | 51(47.7) | 94(87.9) | 88(82.2) | 51(47.7) |
| 80-89 years, No. (%) |  |  |  |  |  |
| Both | 671(87.8) | 401(52.5) | 605(79.2) | 590(77.2) | 491(64.3) |
| Male | 255(86.2) | 143(48.3) | 221(74.7) | 228(77.0) | 212(71.6) |
| Female | 416(88.9) | 258(55.1) | 384(82.1) | 362(77.4) | 279(59.6) |
| 90-99 years, No. (%) |  |  |  |  |  |
| Both | 1229(83.6) | 793(53.9) | 1176(80.0) | 1197(81.4) | 1056(71.8) |
| Male | 429(84.1) | 253(49.6) | 381(74.7) | 409(80.2) | 383(75.1) |
| Female | 651(66.2) | 540(56.2) | 795(82.7) | 788(82.0) | 673(70.0) |
| ≥100 years, No. (%) |  |  |  |  |  |
| Both | 954(78.7) | 647(53.4) | 1067(88.0) | 1076(88.8) | 978(80.7) |
| Male | 171(77.0) | 107(48.2) | 180(81.1) | 188(84.7) | 187(84.2) |
| Female | 783(79.1) | 540(54.6) | 998(89.6) | 888(89.7) | 791(79.9) |

**Supplementary Table 2. Association between pre-frailty/frailty and the risk of multiple adverse outcomes, gender-stratified.**

| Frailty Status | Male | | | Female | | |
| --- | --- | --- | --- | --- | --- | --- |
|  | **Case No.** | **Adjusted OR**  **(95% CI)** | ***P* value** | **Case No.** | **Adjusted OR**  **(95% CI)** | ***P* value** |
| *Incident limited physical performance* | | |  |  |  |  |
| Non-frailty | 1154 | Ref. | Ref. | 871 | Ref. | Ref. |
| Pre-frailty | 2620 | 1.2(1.0-1.4) | 0.123 | 2802 | 1.3(1.1-1.6) | 0.010 |
| Frailty | 464 | 6.9(2.5-18.9) | <0.001 | 880 | 4.7(2.3-9.4) | <0.001 |
| *Incident cognitive decline* | |  |  |  |  |  |
| Non-frailty | 176 | Ref. | Ref. | 226 | Ref. | Ref. |
| Pre-frailty | 491 | 1.3(1.0-1.8) | 0.055 | 786 | 1.4(1.1-1.9) | 0.019 |
| Frailty | 73 | 6.7(2.6-17.1) | <0.001 | 234 | 4.4(2.2-8.8) | <0.001 |
| *Incident dependence* |  |  |  |  |  |  |
| Non-frailty | 175 | Ref. | Ref. | 152 | Ref. | Ref. |
| Pre-frailty | 478 | 1.2(0.9-1.6) | 0.212 | 664 | 1.7(1.3-2.2) | <0.001 |
| Frailty | 99 | 16.6(6.2-44.9) | <0.001 | 349 | 4.9(3.1-8.0) | <0.001 |

Adjusted for: age, gender, education, household income, smoke status, and comorbidity count at baseline.

Abbreviation: OR: odds ratio; CI: confidence interval

**Supplementary Table 3. Association between frailty domains and the risk of mortality, age- and gender-stratified.** Adjusted for: education, household income, smoke status, and comorbidity count at baseline.

| Groups | Exhaustion | | Shrink | | Weakness | | Low Mobility | | Inactivity | |
| --- | --- | --- | --- | --- | --- | --- | --- | --- | --- | --- |
|  | **Adjusted HR**  **(95% CI)** | ***P* value** | **Adjusted HR**  **(95% CI)** | ***P* value** | **Adjusted HR**  **(95% CI)** | ***P* value** | **Adjusted HR**  **(95% CI)** | ***P* value** | **Adjusted HR**  **(95% CI)** | ***P* value** |
| Overall | 1.18(1.12-1.24) | <0.001 | 1.10(1.05-1.15) | <0.001 | 1.62(1.53-1.71) | <0.001 | 1.67(1.58-1.77) | <0.001 | 1.61(1.53-1.70) | <0.001 |
| Male | 1.25(1.17-1.35) | <0.001 | 1.15(1.07-1.24) | <0.001 | 1.80(1.64-1.98) | <0.001 | 1.84(1.68-2.02) | <0.001 | 1.60(1.48-1.74) | <0.001 |
| Female | 1.11(1.03-1.19) | 0.003 | 1.06(0.99-1.13) | 0.078 | 1.51(1.41-1.62) | <0.001 | 1.57(1.46-1.68) | <0.001 | 1.61(1.50-1.73) | <0.001 |
| 65-79 years |  |  |  |  |  |  |  |  |  |  |
| Both | 1.36(1.20-1.54) | <0.001 | 1.26(1.09-1.47) | 0.002 | 2.44(1.95-3.07) | <0.001 | 2.83(2.29-3.50) | <0.001 | 1.87(1.53-2.28) | <0.001 |
| Male | 1.45(1.23-1.70) | <0.001 | 1.12(0.91-1.37) | 0.287 | 2.78(1.99-3.89) | <0.001 | 3.15(2.33-4.24) | <0.001 | 1.48(1.15-1.89) | 0.002 |
| Female | 1.23(1.00-1.51) | 0.049 | 1.46(1.16-1.82) | 0.001 | 2.21(1.62-3.01) | <0.001 | 2.56(1.89-3.47) | <0.001 | 3.50(2.52-4.87) | <0.001 |
| 80-89 years |  |  |  |  |  |  |  |  |  |  |
| Both | 1.17(1.07-1.28) | 0.001 | 1.13(1.03-1.24) | 0.011 | 1.84(1.65-2.04) | <0.001 | 1.90(1.71-2.12) | <0.001 | 1.76(1.59-1.95) | <0.001 |
| Male | 1.22(1.08-1.38) | 0.001 | 1.19(1.05-1.36) | 0.008 | 1.97(1.66-2.33) | <0.001 | 2.11(1.81-2.47) | <0.001 | 1.70(1.48-1.95) | <0.001 |
| Female | 1.10(0.96-1.26) | 0.188 | 1.06(0.93-1.21) | 0.362 | 1.76(1.53-2.02) | <0.001 | 1.75(1.52-2.02) | <0.001 | 1.84(1.59-2.14) | <0.001 |
| 90-99 years |  |  |  |  |  |  |  |  |  |  |
| Both | 1.11(1.02-1.21) | 0.014 | 1.07(0.98-1.16) | 0.117 | 1.56(1.44-1.70) | <0.001 | 1.61(1.48-1.75) | <0.001 | 1.68(1.55-1.83) | <0.001 |
| Male | 1.20(1.06-1.37) | 0.004 | 1.13(1.00-0.29) | 0.055 | 1.67(1.46-1.92) | <0.001 | 1.66(1.46-1.89) | <0.001 | 1.63(1.44-1.85) | <0.001 |
| Female | 1.04(0.93-1.17) | 0.516 | 1.02(0.92-1.14) | 0.671 | 1.50(1.34-1.67) | <0.001 | 1.58(1.42-1.77) | <0.001 | 1.73(1.55-1.94) | <0.001 |
| ≥100 years |  |  |  |  |  |  |  |  |  |  |
| Both | 1.18(1.05-1.32) | 0.005 | 1.03(0.93-1.15) | 0.552 | 1.36(1.22-1.52) | <0.001 | 1.39(1.24-1.55) | <0.001 | 1.30(1.17-1.45) | <0.001 |
| Male | 1.32(1.03-1.68) | 0.028 | 1.10(0.87-1.40) | 0.42 | 1.64(1.29-2.09) | <0.001 | 1.56(1.22-1.98) | <0.001 | 1.39(1.10-1.75) | 0.005 |
| Female | 1.15(1.01-1.32) | 0.033 | 1.01(0.90-1.14) | 0.815 | 1.30(1.15-1.48) | <0.001 | 1.35(1.19-1.53) | <0.001 | 1.27(1.12-1.44) | <0.001 |
